# Leucine Supplementation Does Not Attenuate Skeletal Muscle Loss during Leg Immobilization in Healthy, Young Men

**DOI:** 10.3390/nu10050635

**Published:** 2018-05-17

**Authors:** Evelien M.P. Backx, Astrid M.H. Horstman, Gabriel N. Marzuca-Nassr, Janneau van Kranenburg, Joey S. Smeets, Cas J. Fuchs, Anniek A.W. Janssen, Lisette C.P.G.M. de Groot, Tim Snijders, Lex B. Verdijk, Luc J.C. van Loon

**Affiliations:** 1 Department of Human Biology, NUTRIM School of Nutrition and Translational Research in Metabolism, Maastricht University, P.O. Box 61 6200 MD Maastricht, The Netherlands; emp.backx@gmail.com (E.M.P.B.); astrid_horstman@hotmail.com (A.M.H.H.); g.marzucanassr@maastrichtuniversity.nl (G.N.M.-N.); j.vankranenburg@maastrichtuniversity.nl (J.v.K.); joey.smeets@maastrichtuniversity.nl (J.S.S.); cas.fuchs@maastrichtuniversity.nl (C.J.F.); tim.snijders@maastrichtuniversity.nl (T.S.); Lex.verdijk@maastrichtuniversity.nl (L.B.V.); 2 Department of Human Nutrition, Wageningen University, P.O. Box 17 6700 AA Wageningen, The Netherlands; lisette.degroot@wur.nl; 3 Department of Surgery, Maastricht University Medical Centre+, P.O. Box 616 6200 MD Maastricht, The Netherlands; anniek.janssen@mumc.nl

**Keywords:** limb immobilization, countermeasures, amino acid, strength

## Abstract

Background: Short successive periods of physical inactivity occur throughout life and contribute considerably to the age-related loss of skeletal muscle mass. The maintenance of muscle mass during brief periods of disuse is required to prevent functional decline and maintain metabolic health. Objective: To assess whether daily leucine supplementation during a short period of disuse can attenuate subsequent muscle loss in vivo in humans. Methods: Thirty healthy (22 ± 1 y) young males were exposed to a 7-day unilateral knee immobilization intervention by means of a full leg cast with (LEU, *n* = 15) or without (CON, *n* = 15) daily leucine supplementation (2.5 g leucine, three times daily). Prior to and directly after immobilization, quadriceps muscle cross-sectional area (computed tomography (CT) scan) and leg strength (one-repetition maximum (1-RM)) were assessed. Furthermore, muscle biopsies were taken in both groups before and after immobilization to assess changes in type I and type II muscle fiber CSA. Results: Quadriceps muscle cross-sectional area (CSA) declined in the CON and LEU groups (*p* < 0.01), with no differences between the two groups (from 7712 ± 324 to 7287 ± 305 mm^2^ and from 7643 ± 317 to 7164 ± 328 mm^2^; *p* = 0.61, respectively). Leg muscle strength decreased from 56 ± 4 to 53 ± 4 kg in the CON group and from 63 ± 3 to 55 ± 2 kg in the LEU group (main effect of time *p* < 0.01), with no differences between the groups (*p* = 0.052). Type I and II muscle fiber size did not change significantly over time, in both groups (*p* > 0.05). Conclusions: Free leucine supplementation with each of the three main meals (7.5 g/d) does not attenuate the decline of muscle mass and strength during a 7-day limb immobilization intervention.

## 1. Introduction

The progressive decline of skeletal muscle mass and strength is one of the hallmarks of aging. This loss of muscle mass and strength leads to a reduction in functional capacity and increases the risk of development of chronic metabolic diseases [1]. Though the mechanisms responsible for age-related muscle mass loss remain to be elucidated, it is clear that an inadequate protein intake and a reduced level of physical activity play a key role [2]. Recently, it has been suggested that age-related muscle loss may be largely attributed to the muscle loss that manifests during brief successive periods of muscle disuse throughout the lifespan [3,4,5]. Merely a few days of immobilization [6,7,8,9,10] or bedrest [11,12,13] can result in substantial muscle and strength loss in older individuals. Furthermore, it has been reported that older individuals have a reduced capacity to regain the skeletal muscle mass that was lost during a period of disuse [14]. As a consequence, our group and others have started to look for strategies to attenuate or even avoid muscle loss during brief periods of muscle disuse to support healthy aging.

Muscle mass is regulated by fluctuations in muscle protein synthesis and breakdown rates [15,16]. The loss of muscle mass during a period of disuse has been attributed to both a decline in basal muscle protein synthesis [11,17,18,19,20,21] and a diminished muscle protein synthetic response to protein ingestion [21,22,23,24]. Furthermore, a rapid increase in muscle protein breakdown rate has been suggested to occur during the early stages of muscle disuse [2,25,26,27]. Protein ingestion forms a key anabolic stimulus with a fast increase in muscle protein synthesis rates and an inhibition of proteolysis following the post-prandial rise in plasma amino acid concentrations. It has been well established that the post-prandial increase in muscle protein synthesis rate is attributed to the rise in plasma essential amino acids concentrations [28,29], with leucine being the most relevant [30,31,32,33,34,35]. Leucine administration has been shown to stimulate muscle protein synthesis by direct or indirect activation of many key regulatory proteins in the mTOR pathway [36,37,38,39]. Furthermore, leucine has been reported to have strong anti-proteolytic properties [40], either directly or via its insulinotropic properties [41,42]. We hypothesize that providing additional free leucine with each main meal may compensate for anabolic resistance and, as such, avoid or diminish muscle mass loss during a brief period of immobilization.

In the present study, we subjected 30 healthy, young men to 7 days of unilateral knee immobilization. One group (*n* = 15) was supplemented with 2.5 g of leucine three times daily during immobilization (7.5 g/d in total), whereas the other group (*n* = 15) ingested a control supplement (15 g glucose plus maltodextrin). 

## 2. Methods

### 2.1. Subjects

The study was performed as part of a greater project in which we also investigated the impact of creatine supplementation on muscle mass loss (NIH Clinical Trial Registration Number: NCT01894737) [43]. Thirty young (18–35 years) healthy males (with a body mass index (BMI) between 18.5–30 kg/m^2^) participated in this randomized controlled trial. Participants’ characteristics are presented in Table 1. All subjects were screened and excluded based on exclusion criteria described previously [43]. All participants were informed about the purpose of the study, the experimental procedures, and all its possible risks prior to providing written consent to participate. This study was approved by the Medical Ethics Committee from Maastricht University Medical Center+ (MUMC+). The procedures followed were in accordance with the ethical standards of the responsible institutional or regional committee on human experimentation or in accordance with the Declaration of Helsinki of 1975 as revised in 1983. 

### 2.2. Pretesting/Screening

Body weight and height were measured using a digital balance and a stadiometer to calculate the BMI. A medical questionnaire was filled in to assess whether the subjects met the inclusion criteria. Subsequently, a familiarization session on the leg extension machine (Technogym, Rotterdam, The Netherlands) was performed as described previously [44,45].

### 2.3. Experimental Outline

After inclusion into the study, the subjects were subjected to 7 days of muscle disuse induced by means of a full leg cast. During the immobilization period, the subjects received daily leucine supplementation (LEU, *n* = 15). A comparison was made with a control group receiving daily placebo supplementation (CON, *n* = 15). The data from the CON group have been published previously [43]. The study procedures were identical for both groups: the immobilized leg was randomly allocated and counterbalance between left and right was ensured. Two days prior to casting and directly after cast removal, a series of measurements were performed. Single-slice computed tomography (CT) scans were performed at the mid-thigh of both legs. In addition, a single muscle biopsy from the immobilized leg and a venous blood sample were collected. Leg extension strength was assessed (1 repetition maximum) for both legs separately. All analyses were performed by investigators blinded to subject coding.

### 2.4. Measurements

The subjects participated in two identical experimental test days, before and immediately after the immobilization period. Two days prior to the immobilization period, the subjects arrived at the laboratory at 8.00 a.m. after an overnight fast, and their body weight was measured with a digital balance with an accuracy of 0.1 kg (SECA GmbH, Hamburg, Germany). Thereafter, a single-slice CT scan (Philips Brilliance 64; Philips Medical Systems, Best, The Netherlands) was performed to determine quadriceps muscle and whole thigh muscle cross-sectional area (CSA) in both legs, as described previously [43]. Subsequently, a blood sample was taken from the antecubital vein. After collection, the EDTA-containing blood tubes were immediately centrifuged at 1000× *g* for 10 min at 4 °C. Aliquots of plasma were frozen in liquid nitrogen and stored at −80 °C until analysis.

Thereafter, a muscle biopsy was collected from the leg that was subsequently immobilized by using the percutaneous needle biopsy technique [46], as described previously [43]. 

After consuming breakfast, each subject’s single-leg one repetition maximum (1-RM) was assessed on a leg extension machine. The estimations obtained during the screening visit were used to determine 1-RM [45]. The subjects were familiarized with, and instructed on, the usage of crutches on the second test day, post-immobilization. Finally, the cast was removed, and the measurements described above (CT scan, muscle biopsy, blood draw, and 1-RM leg strength) were performed.

### 2.5. Limb Immobilization Protocol

Two days after the first test day, the subjects reported at 8:00 a.m. at the Casting Room at Maastricht University Medical Centre, to have a full leg cast fitted. The casting procedure has been described previously [44,47]. 

### 2.6. Leucine Supplementation

Subjects in the LEU group were supplemented with free crystalline leucine (BUFA, Uitgeest, The Netherlands) for one week, starting at the first day of immobilization. The leucine was provided in capsules (0.5 g free leucine per capsule). The supplementation protocol consisted of 7.5 g leucine per day, i.e., 2.5 g with each main meal (breakfast, lunch, and dinner) during the immobilization week, based upon previous published research [48,49]. The control group received a placebo supplement consisting of 7.5 g maltodextrin and 7.5 g dextrose monohydrate (AVEBE, Veendam, The Netherlands). 

### 2.7. Dietary Intake and Physical Activity Records

To limit the potential impact of food ingestion on blood and/or muscle biopsy outcome parameters, the subjects received a standardized meal containing 2.9 MJ providing 51 energy% (En%) as carbohydrate, 32 En% as fat, and 17 En% as protein, on the evening prior to both test days. The subjects were asked to maintain their habitual food intake during the study and to refrain from consuming alcohol in the 48 h leading up to a test day. Two weekdays and one weekend day dietary intake records were completed before and during the immobilization period. The dietary intake records were analyzed using dieetInzicht software, based on the Dutch food composition table (NEVO table) 2011. The dietary intake records were excluded if they were presented empty, or if the overall energy consumption was lower than 750 kcal per day. The participants refrained from any sort of exhaustive physical exercise from 48 h before the first test until the end of the study.

### 2.8. Blood Analyses

Plasma leucine concentrations were determined by gas chromatography–mass spectrometry (GC–MS) (Agilent 7890A GC/5975C; MSD, Little Falls, DE, USA). The internal standard [U-^13^C_6_]-Leucine was added to the samples. The plasma samples were deproteinized on ice with dry 5-sulfosalicylic acid. Before analysis by GC–MS, leucine was purified using cation exchange AG 50W-X8 resin (mesh size: 100–200, ionic form: hydrogen (Bio-Rad Laboratories, Hercules, CA, USA)) columns and converted to its tert-butyl dimethylsilyl (TBDMS) derivative. Electron impact ionization by monitoring ions at mass/charge (*m/z*) 302 and 308 for unlabelled and [U-^13^C_6_]-Leucine, respectively, was used to determine leucine concentration. Standard regression curves were applied from a series of known standard concentration values against the measured values to assess the linearity of the mass spectrometer measures. 

### 2.9. Muscle Analyses 

Immunohistochemistry was performed to determined type I and type II muscle fiber CSA on 5-µm-thick muscle biopsy cryosections. The staining and recording procedures were performed as described previously [50]. Pre and post-immobilization muscle biopsy samples were successfully collected of sufficient quality to assess muscle fiber characteristics in 24 participants (*n* = 13 in CON group, *n* = 11 in LEU group). The investigators were blinded to subject coding during the analysis. The mean muscle fiber size was calculated for the type I and type II muscle fibers separately. Form factors were calculated by using the following formula: (4π·CSA)/(perimeter)^2^ to assess fiber circularity. No change over time or between groups were observed for fiber circularity. The mean number of fibers included in the analyses was 274 ± 25 and 306 ± 28 for pre- and post-immobilization samples, respectively.

### 2.10. Statistics

All data are expressed as mean ± SEM. An independent samples *t*-test was used to assess baseline differences between groups. Repeated measures ANOVA with treatment (CON versus LEU) as between-subject factor and time (pre- versus post-immobilization) as within-subject factor was used to analyze pre- versus post-immobilization data. The within-subjects factor “fiber type” (type I versus type II) was added to the statistical analyses for the muscle fiber data. A *p*-value <0.05 was used to determine the significance. SPSS version 22.0 (SPSS, IBM Corp., Armonk, NY, USA) was used to analyze the data.

## 3. Results

### 3.1. Subjects

The subjects’ characteristics are provided in Table 1. The mean age of the subjects was 22 ± 1 y with an average BMI of 23.1 ± 0.5 kg·m^−2^. None of the baseline characteristics were different between the CON and LEU groups. Because of time constraints, three participants withdrew prior to immobilization. From the 30 participants, 27 completed the study (CON: *n* = 13; LEU: *n* = 14). 

### 3.2. Dietary Intake

The baseline habitual energy intake was 8.5 ± 0.5 MJ/d and did not change significantly over time (*p* ≥ 0.05; Table 2). No significant interaction for energy intake was found over time between the groups. The average habitual protein intake prior to immobilization was 1.2 ± 0.1 and 1.3 ± 0.1 g/kg/d in the CON and LEU groups, respectively, with no significant changes over time (*p* ≥ 0.05; Table 2).

### 3.3. Muscle Mass and Strength 

**Figure 1:**
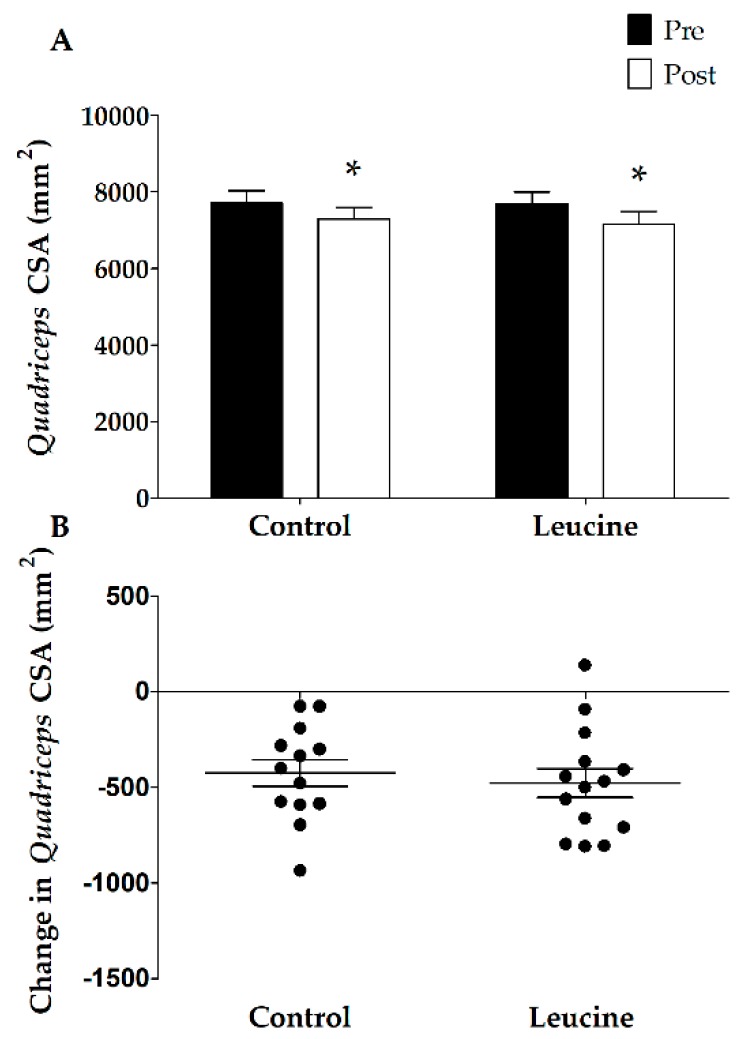
(**A**) Cross-sectional area (CSA) of the quadriceps muscle in the control (*n* = 13) and leucine (*n* = 14) groups before and after 7 days of unilateral knee immobilization. (**B**) Individual changes in quadriceps muscle CSA following 7 days of one-legged knee immobilization. The data were analyzed using repeated measures ANOVA. The data are expressed as means ± SEM. Immobilization resulted in a significant decline in quadriceps muscle CSA in both groups (*), with no differences between the groups.

**Figure 2:**
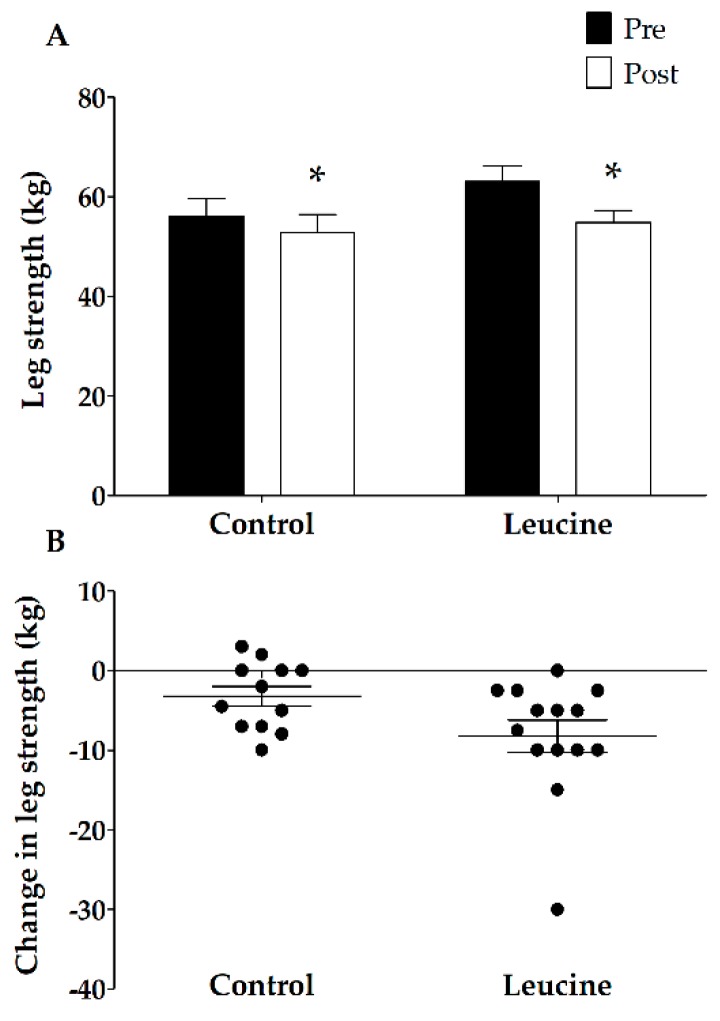
(**A**) One-repetition maximum (1-RM) leg muscle strength in the control (*n* = 13) and leucine (*n* = 14) group before and after 7 days of unilateral knee immobilization. (**B**) Individual changes in 1-RM leg muscle strength following 7 days of one-legged knee immobilization. The data were analyzed using repeated measures ANOVA. The data are expressed as means ± SEM. Immobilization resulted in a significant decline in 1-RM leg muscle strength in both groups (*), with no differences between the groups.

Before the intervention, no differences were observed in quadriceps muscle CSA between the groups (*p* = 0.88; Figure 1)*.* Seven days of immobilization caused a significant reduction in quadriceps muscle CSA (time effect *p* < 0.01; from 7712 ± 324 to 7287 ± 305 mm^2^ versus 7643 ± 317 to 7164 ± 328 mm^2^ in the CON and LEU groups, respectively). No differences were observed between the CON and LEU groups (interaction effect *p* = 0.61). One-repetition maximum leg muscle strength decreased significantly during immobilization from 56 ± 4 kg to 53 ± 4 kg in the CON group and from 63 ± 3 kg to 55 ± 2 kg in the LEU group (main effect of time *p* < 0.01; Figure 2). No significant differences were observed between the CON and LEU groups (interaction effect; *p* = 0.052; Figure 2).

### 3.4. Plasma Analyses

Fasting plasma leucine concentrations did not change in the LEU (154 ± 5 and 160 ± 4 μmol·L^−1^ before and after supplementation, respectively) and CON groups (156 ± 5 and 150 ± 6 μmol·L^−1^ before and after supplementation, respectively). No differences in fasting plasma leucine concentrations were observed between the CON and LEU groups (*p* = 0.13).

### 3.5. Muscle Fiber Characteristics

In the baseline muscle biopsy sample, no differences were observed for any of the variables between the two groups (Table 3). No significant changes in muscle fiber CSA were observed following 7 days of immobilization (*p* ≥ 0.05). Muscle fiber type distribution (in % and % area) did not change significantly following 7 days of immobilization (*p* ≥ 0.05), and no differences were observed between the groups (*p* ≥ 0.05). Muscle fiber CSA and fiber type distribution (in % and % area) were different between type I and type II muscle fibers at all time points (*p* ≤ 0.01).

## 4. Discussion

The present study demonstrates that fortification of the main meals with free leucine did not attenuate muscle disuse atrophy or muscle strength loss during seven-day unilateral knee immobilization in healthy adults. 

Skeletal muscle loss experienced during brief successive periods of muscle disuse (e.g., following injury or during recovery from illness) has been hypothesized to be largely accountable for the loss of muscle mass throughout the lifespan [4,5,47]. Most periods of forced muscle disuse are of relative brief duration, lasting less than one week [4,51]. Whereas ample studies have shown that long-term disuse leads to substantial muscle loss [18,52,53,54], only a limited number of short-term disuse studies have been performed [44,55]. In the current study, we show that as little as seven days of muscle disuse already leads to a considerable loss of muscle mass. We observed a 6% decline in quadriceps muscle CSA (Figure 1), which was accompanied by a 9% decline in leg strength (Figure 2) in healthy males. These findings are in line with previous work from our group [4,10,44,47] as well as others [56] and underline the impact of even short periods of muscle disuse on muscle mass and strength. Disuse atrophy is accompanied by many negative health consequences, such as reduced functional capacity, increased risk of post-surgery complications and co-morbidities, and less successful rehabilitation upon hospital discharge [20,57,58,59,60]. Clearly, there is a need for effective strategies to avoid or attenuate the decline in skeletal muscle mass or strength during brief periods of muscle disuse in both health and disease.

The decline of skeletal muscle mass during a period of physical activity has been, at least partly, attributed to a reduction in the muscle protein synthetic response to meal ingestion, now coined anabolic resistance [21,61]. Increasing protein intake [62] or co-ingesting free leucine [48,63] has previously been shown to increase the post-prandial muscle protein synthetic response to protein ingestion, implying that free leucine supplementation or food fortification with leucine may represent effective strategies to compensate for anabolic resistance and, as such, to attenuate muscle mass loss [49]. Consequently, we hypothesized that free leucine supplementation with each main meal (3 × 2.5 g per day) compensates for anabolic resistance and attenuates muscle mass loss during seven days of muscle disuse. However, the present data showed no differences in leg muscle loss between the leucine and control groups (−479 ± 75 and −424 ± 69 mm^2^, respectively; *p* = 0.61). In line with the loss of muscle mass, muscle strength loss did not differ significantly between the groups (−8.2 ± 2.0 and −3.2 ± 1.2 kg in the leucine and control groups, respectively; *p* = 0.052). 

A recent study from English et al. determined the impact of leucine supplementation during two weeks of bedrest in young individuals [64]. Their study seemed to support our hypothesis, as leucine supplementation (4.4 g leucine per meal, 13.2 g per day) attenuated lean mass loss following seven days of bedrest (−1.5 ± 0.3 and −0.8 ± 0.3 kg lean mass loss in the control and leucine groups, respectively). However, the attenuated loss of lean mass was no longer evident following two weeks of bedrest (−1.5 ± 0.3 and −1.0 ± 0.3 kg lean mass loss, respectively). Our data show no benefits of leucine in preserving either muscle mass or strength following one week of limb immobilization. Whether the apparent discrepancy between our data and those by English et al. is to be attributed to the disuse model used (limb immobilization versus bedrest) or the amount of leucine provided (7.5 versus 13.2 g per day) remains unclear. Nonetheless, our work is in line with previous work by our group, as we have been unable to detect net changes in muscle mass, strength, or glucose homeostasis following three and six months of leucine supplementation in healthy individuals [65] and type 2 diabetes patients [66]. In the latter study, we hypothesized that free leucine supplementation may be more relevant under more clinically compromised conditions where muscle atrophy is apparent. However, on the basis of the present data, we can only conclude that under well-fed conditions, a local anabolic resistance due to limb immobilization [24] cannot be compensated for by additional free leucine ingestion. We need to stress that, in our study, healthy subjects were fed in energy balance, with an (overall) average dietary protein intake of 1.2 ± 0.1 g protein·kg^−1^ body weight per day, which is well in excess of the Recommended Dietary Allowance (RDA) [67]. As dietary protein intake does not meet the RDA in a large proportion of institutionalized older people or more clinically comprised individuals admitted to hospital [68], these results may not necessarily represent the impact of leucine supplementation on preserving skeletal muscle mass during disuse under malnourished conditions. 

## 5. Conclusions

In conclusion, free leucine ingestion with each main meal (3 × 2.5 g leucine per day) does not attenuate the loss of leg muscle mass and leg muscle strength during a brief period of unilateral knee immobilization in healthy, young males. 

## Figures and Tables

**Table 1:** Subjects’ characteristics.

	Control	Leucine
Age (years)	23 ± 1	21 ± 1
Body mass (kg)	73.1 ± 3.2	73.5 ± 1.9
Height (m)	1.76 ± 0.03	1.80 ± 0.02
BMI (kg·m^−2^)	23.5 ± 0.8	22.7 ± 0.6
1 RM leg extension (kg)	56 ± 4	63 ± 3
Whole-thigh muscle CSA (mm^2^)	14,184 ± 462	14,417 ± 537
Quadriceps muscle CSA (mm^2^)	7712 ± 324	7643 ± 317

The values are means ± SEMs. Abbreviations: BMI = Body Mass Index, CSA = Cross-sectional area, 1 RM: one-repetition maximum. The baseline characteristics were not different between the groups.

**Table 2:** Habitual dietary intake in the control and leucine groups.

	Control Group (*n* = 14)		Leucine Group (*n* = 8)	
	Pre-Immobilization	During Immobilization	Pre-Immobilization	During Immobilization
Energy (MJ/day)	7.6 ± 0.4	7.4 ± 0.6	10.0 ± 1	9.8 ± 1
Carbohydrate (En%)	54 ± 8	47 ± 3	52 ± 3	50 ± 3
Fat (En%)	31 ± 2	31 ± 3	31 ± 3	29 ± 3
Protein (En%)	19 ± 1	18 ± 1	16 ± 1	17 ± 2
Protein (g/kg/day)	1.2 ± 0.1	1.1 ± 0.1	1.3 ± 0.2	1.3 ± 0.2

The values are means ± SEMs. Abbreviation: En% = Energy%. The data are expressed as means ± SEM. The data were analyzed using repeated measures ANOVA. No differences between the leucine and control groups were found over time.

**Table 3:** Muscle fiber characteristics in the control and leucine groups.

Fiber Type		Control Group (*n* = 13)		Leucine Group (*n* = 11)	
		Pre-Immobilization	Post-Immobilization	Pre-Immobilization	Post-Immobilization
Muscle fiber CSA (μm2)	**I**	6034 ± 501	6620 ± 508	5781 ± 354	6196 ± 415
	**II** *	7202 ± 640	7540 ± 587	6033 ± 482	6589 ± 509
Fiber type distribution (%)	**I**	38 ± 4	33 ± 3	39 ± 5	41 ± 5
	**II** *	62 ± 4	67 ± 3	61 ± 5	59 ± 5
Fiber type distribution (% area)	**I**	34 ± 4	31 ± 3	38 ± 4	40 ± 5
	**II** *	66 ± 4	69 ± 3	62 ± 4	60 ± 5

The values are means ± SEMs. CSA, cross-sectional area. No differences were found between the control and leucine groups for all variables at all time points. * Muscle fiber CSA and fiber type distribution (in % and % area) were different between type I and type II muscle fibers at all time points, *p* < 0.05.

## Abbreviations

1-RM	one-repetition maximum
BSA	bovine serum albumin
CON	control group
CSA	cross-sectional area
CT-scan	computed tomography-scan
EDTA	ethylenediaminetetraacetic acid
FT	fiber typing
LEU	leucine group
MHC	myosin heavy chain
RDA	Recommended Dietary Allowance
TBDMS	tert-butyl dimethylsilyl
